# Visual Acuity Correlations of Preoperative OCT Morphological Parameters After Phacovitrectomy for Epiretinal Membrane

**DOI:** 10.1155/joph/8892409

**Published:** 2026-02-13

**Authors:** Zhmurin R., Grajewski L., Krause L.

**Affiliations:** ^1^ Department of Ophthalmology, Staedtisches Klinikum Dessau, Brandenburg Medical School Theodor Fontane, Dessau, Germany; ^2^ Office of Doctoral Studies, Charité—Universitaetsmedizin Berlin, Berlin, Germany

**Keywords:** epiretinal membrane, OCT morphological parameters, phacovitrectomy

## Abstract

**Background/Aims:**

The aim of this study was to demonstrate a possible correlation between qualitative preoperative OCT morphological changes in epiretinal membrane (ERM) and best corrected visual acuity (BCVA).

**Methods:**

A total of 201 patient eyes with idiopathic symptomatic ERM and cataracts were included in the retrospective study. Phacovitrectomy was performed between 2015 and 2019.

**Results:**

ERM was classified into five OCT morphotypes. The first three corresponded to the ERM classification according to Govetto et al., Stages 1–3 without a tractional component, while the last two each had a tractional component in the sense of a lamellar macular hole and vitreomacular traction. A significant difference in preoperative BCVA was observed between all OCT morphotypes (*p* < 0.01). For OCT morphotypes without a tractional component, a negative correlation (*p* < 0.05) was observed between postoperative BCVA and lesions of the outer foveal layers (damaged retinal pigment epithelium, disrupted ellipsoid zone, and external limiting membrane). Nonedematous lesions of the inner foveal layers (detachment of the outer nuclear layer, depth of retinal layer distortion, breakage and distortion of retinal nerve fiber layers, and ERM separation) showed no correlation with either preoperative or postoperative BCVA in all OCT morphotypes (*p* > 0.05). Likewise, edematous changes in the inner foveal layers played no role in pre‐ and postoperative visual acuity prognosis (*p* > 0.05).

**Conclusion:**

Preoperative OCT morphological changes in the inner foveal layers have no significance in postoperative visual acuity prognosis in ERM, in contrast to lesions of the outer central retinal layers.

## 1. Introduction

Epiretinal membrane (ERM) is a progressive macular disease associated with avascular hemitransparent fibrocellular proliferation across the inner limiting membrane (ILM) on the retinal surface, involving the fovea [1, 2]. Etiologically, ERM can be divided into two forms: the common idiopathic form (primary, approximately 4/5 of all cases) with histopathologically evident proliferation of myofibroblasts, fibroblasts, hyalocytes, and retinal glial cells (Müller cells), and the much rarer secondary form [2, 3]. The latter is partly associated with the flushing of retinal pigment epithelial (RPE) cells as well as with macrophage activation in inflammatory, vascular and tumorous eye diseases as well as by retinal foramina, blunt and perforating trauma, laser or cryocoagulation, and globe penetrating surgical procedures of any kind [1]. Idiopathic ERM, including cellophane maculopathy, typically occurs from the age of 50 onward. Its prevalence correlates with age, female sex, the presence of diabetes mellitus, and hypercholesterolemia [2], with an incidence of approximately 12% depending on the population at the age of 70–80 years [4, 5]. It is worth noting that cataracts, the most common age‐related pathology of the anterior segment of the eye, are similar in prevalence to ERM. Furthermore, ERM is often accompanied by cataracts at this age. The main symptoms of ERM include increasing metamorphopsia and the resulting visual impairment. Pathophysiologically, myofibroblast‐induced retinal contraction with the resulting tangential retinal traction forces, as well as the development of the tractional component in the case of a lamellar macular hole (LMH), are associated with the aforementioned functional disorders [6].

Native, noninvasive, high‐resolution macular imaging using optical coherence tomography (OCT) is essential for the diagnosis and morphological visualization of ERM. OCT provides valuable information, replacing histopathology, on the qualitative parameters of the individual macular layers, some of which have a reliable prognostic significance for visual acuity. In particular, the role of the intact connection between the inner and outer segments of the photoreceptors (PR, IS/OS junction) has been demonstrated [7]. ERM is classified according to OCT morphology according to Govetto et al. [8] into the following stages: preserved (stage 1) and abolished (stage 2) foveal depression, ectopic inner foveal layers (EIFL, stage 3), and markedly disorganized inner retinal layers (DRIL, stage 4). A special OCT morphological form of ERM includes its combination with an LMH.

The latter is pathophysiologically divided into two types: degenerative, resulting from the development of superficial retinal traction forces in epiretinal cell proliferation [9], and tractional, resulting from the additional development of vitreoretinal traction with typical OCT markers such as a tractional membrane in the vitreous, foveal schisis, intraretinal pseudocysts in tractional macular edema, and a cotton ball sign [10]. There is also the combination of ERM with vitreomacular traction (VMT).

The modern surgical treatment for symptomatic ERM with concurrent cataract is phacovitrectomy, which combines phacoemulsification and IOL implantation with minimally invasive pars plana vitrectomy, membraneectomy, peeling of the ILM, and subsequent air or SF_6_ gas filling. This complex surgical procedure allows the prevention of rapid cataract development in phakic eyes following pars plana vitrectomy and gas filling alone. It is significantly more beneficial for patients due to faster postoperative recovery compared to a two‐stage procedure, as well as for cost savings for the healthcare system. Several studies have demonstrated that phacovitrectomy is not inferior to consecutive surgical interventions on the anterior and posterior segments of the eye in terms of postoperative morphofunctional findings [11] and complication rates [12, 13]. However, despite rapid technical diagnostic and surgical advances, the accurate prognosis of postoperative visual outcomes in ERM based on OCT morphology remains questionable. This partly complicates both preoperative information and postoperative doctor–patient trust, although some predictive morphological biomarkers such as the OI/OS junction [14] and EIFL [8] are described in the publications.

The aim of this retrospective study with a shortened follow‐up period was to determine possible correlations between qualitative preoperative OCT morphological changes of the individual foveal layers in ERM and best‐corrected decimal visual acuity (BCVA) after phacovitrectomy. At the time of follow‐up, the largest postoperative visual acuity improvement was observed after 3 months, with slight improvement over the following years after pars plana vitrectomy in ERM [15].

## 2. Patients and Methods

The study included the findings of 180 patients, 63 males, and 117 females, aged 74.9 ± 6.4 years, with a total of 201 eyes, 100 right, and 101 left eyes. Exclusion criteria were any type of intraocular surgery, severe trauma, inflammation, and other diseases. All patients had symptomatic idiopathic ERM and concurrent low‐grade cataracts. Between 2015 and 2019, all patient eyes underwent combined phacovitrectomy under general anesthesia by two surgeons.

During the preoperative examination, biometry was calculated using the SRK/T formula; for axial lengths of less than 21 mm, the HAIGIS formula was also used. After consultation, all patients desired emmetropia with a residual correction of −0.1 to −0.4 diopters. After phacoemulsification of the lens nucleus, aspiration of the cortical masses and polishing of the posterior capsule, monofocal acrylic posterior chamber lenses with an optic diameter of 6 mm were implanted. Subsequently, a 25 G pars plana vitrectomy with detachment of the posterior vitreous membrane, dye‐assisted membranectomy, ILM peeling, and administration of SF_6_ gas or air was performed. The dyes used were either Membrane Blue (MEMBRANEBLUE DUAL from DORC) in 96 eyes or Brilliant Peel (Brilliant Peel syringe from Geuder) in 105 eyes. Air endotamponade was generally used; in cases of retinal foramina discovered intraoperatively, SF_6_ gas tamponade was always performed after exocryogenic or endolaser coagulation.

The preoperative examination took place 3.6 ± 1.1 days before surgery. The postoperative follow‐up examination took place 3.8 ± 1.2 months before surgery to rule out postoperative complications such as retinal detachment, macular edema, etc. Further examinations were performed by the outpatient ophthalmologists. Furthermore, a maximum increase in BCVA was shown 3 months postoperatively in the treatment of ERM in another study [15].

Decimal BCVA was determined pre‐ and postoperatively, with the corresponding differences calculated. Objective refraction was determined using the Canon R‐F10 Operation Manual autorefractometer, and subjective refraction was determined using the HAAG‐STREIT Möller‐Wedel optotype system with an M 3000 projector. OCT morphological findings for verification of ERM were acquired using the high‐resolution multimodal OCT Spectralis Spec‐CAM‐06961 imaging platform from Heidelberg Engineering.

**FIGURE 1:**
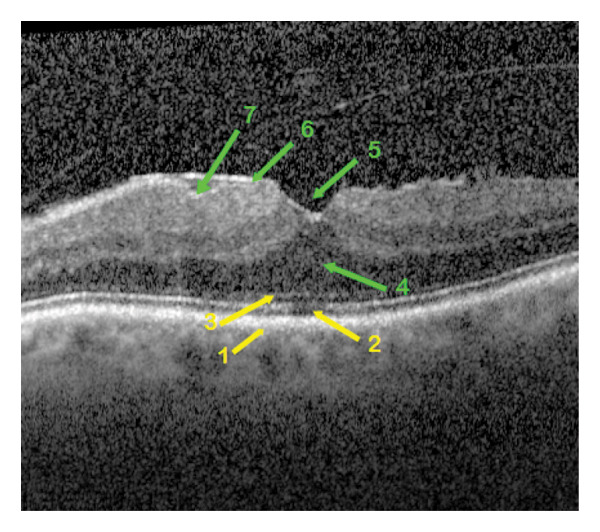
OCT morphological image of epiretinal membrane in a patient eye of group 1 with partially preserved foveal depression. The retinal changes are indicated by arrows of different staining, yellow for the outer retinal layers and green for the inner ones. The characteristics of the foveal layers: 1—intact retinal pigment epithelium, 2—interrupted ellipsoid zone, 3—unchanged external limiting membrane, 4—detached outer nuclear layer, 5—pseudohole, 6—adherent epiretinal membrane with maximum macular thickness nasally, 7—wrinkling of the retinal nerve fiber layer without its rupture.

**FIGURE 2:**
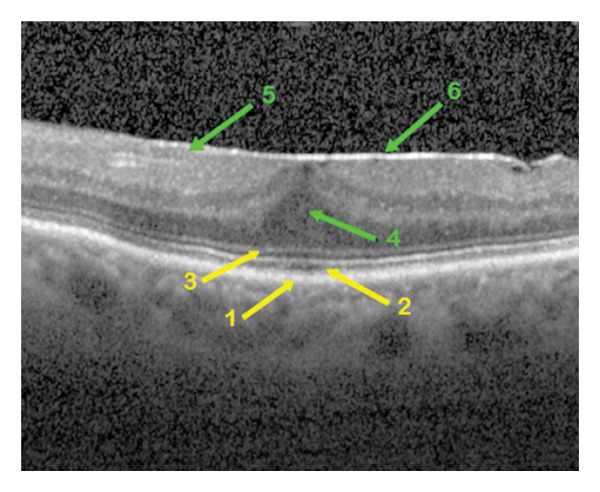
OCT morphological image of the epiretinal membrane in a patient eye of group 2 with isolated foveolar detachment. The retinal changes are indicated by arrows of different coloration, yellow for the outer retinal layers and green for the inner ones. The characteristics of the foveal layers: 1—intact retinal pigment epithelium, 2—optically normal ellipsoid zone, 3—unchanged external limiting membrane, 4—detached outer nuclear layer, 5—intact retinal nerve fiber layer without folds or breaks, 6—adherent epiretinal membrane.

**FIGURE 3:**
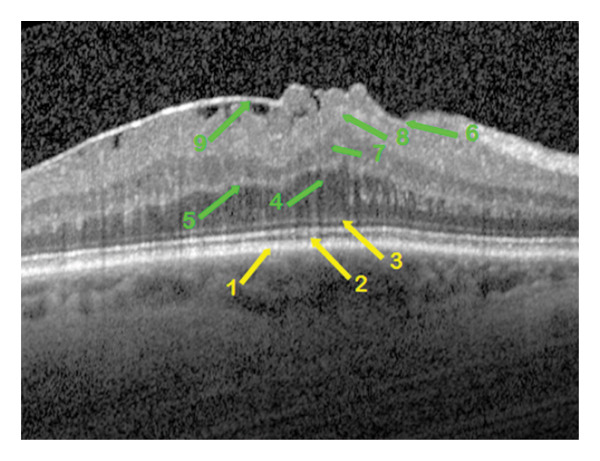
OCT morphological image of the epiretinal membrane in a patient eye of group 3 with ectopic inner retinal layers. The retinal changes are indicated by arrows of different staining, yellow for the outer retinal layers and green for the inner ones. Foveal layer features: 1—intact retinal pigment epithelium, 2—disrupted ellipsoid zone, 3—unchanged external limiting membrane, 4—detached outer nuclear layer with individual barely visible pseudomicrocysts, 5—wrinkling of the outer plexiform layer, 6—wrinkling of the retinal nerve fiber layer without its break, 7—ectopic inner nuclear layer, 8—ectopic ganglion cell layer, 9—separated epiretinal membrane with maximum macular thickness temporally.

**FIGURE 4:**
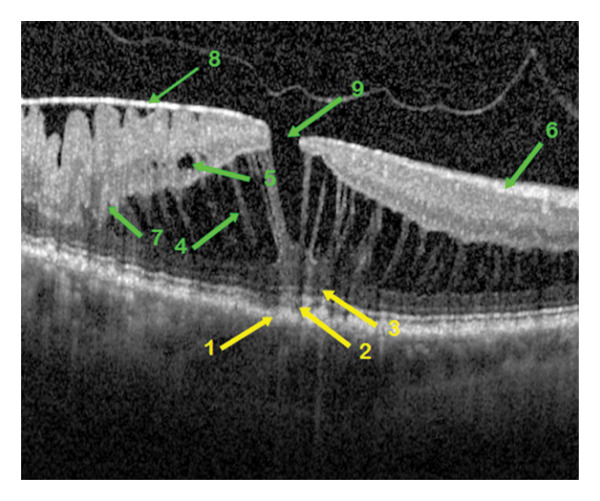
OCT morphological image of the epiretinal membrane in a patient eye of group 4 with a lamellar macular hole of tractional origin. The retinal changes are indicated by arrows of different coloration, yellow for the outer retinal layers and green for the inner retinal layers. The characteristics of the foveal layers: 1—undulating retinal pigment epithelium with the formation of fine drusen, 2—discontinuous ellipsoid zone, 3—undulating external limiting membrane, 4—detached outer nuclear layer with multiple large pseudomicrocysts and suspicious nasal foveal schisis, 5—pseudomicrocysts in the inner nuclear layer, 6—intact retinal nerve fiber layer, 7—wrinkling of the outer plexiform layer, 8—well‐separated epiretinal membrane with maximum macular thickness temporally, 9—tractional lamellar macular hole with visible posterior vitreous detachment.

**FIGURE 5:**
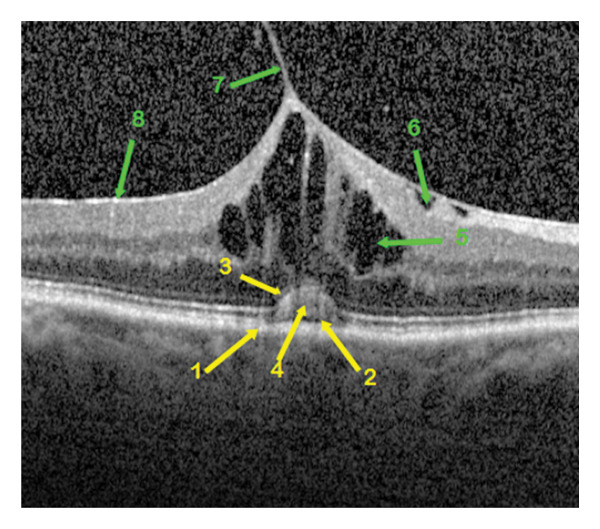
OCT morphological image of the epiretinal membrane in a patient eye of group 5 with vitreomacular traction. The retinal changes are indicated by arrows of different staining, yellow for the outer retinal layers and green for the inner ones. The features of the foveal layers: 1—intact retinal pigment epithelium, 2—discontinuous ellipsoid zone, 3—nasally discontinuous external limiting membrane, 4—cotton ball sign, 5—detached outer nuclear layer with multiple large pseudomicrocysts, 6—wrinkling and rupture of the retinal nerve fiber layer, 7—tractional membrane, 8—adherent epiretinal membrane with maximum macular thickness nasally.

In the central foveal section with a diameter of up to approximately 3 mm, the qualitative OCT parameters of the individual retinal layers as well as the overall ERM morphology were analyzed layer by layer. In all patient eyes, the ERM could be divided into five OCT morphotypes (Figures 1, 2, 3, 4, and 5):

• Group 1. Completely or partially preserved foveolar depression (Figure 1)
• Group 2. Isolated elevation of the foveola (Figure 2)
• Group 3. Elevation of the foveola with ectopia of the inner nuclear layer (INL) and/or the plexiform granular layer (IPL) (EIFL) (Figure 3)
• Group 4. LMH of tractional origin with the OCT markers described above (Figure 4)
• Group 5. VMT (Figure 5)

The first three OCT morphotypes of ERM corresponded to the classification of Govetto et al. [8] and contained no visible tractional OCT biomarkers, but cotton ball signs in approximately one‐fifth of the patient eyes. The last two are pathophysiologically associated with the presence of a tractional component (Figures 4 and 5). Since the number of patients with stage 4 disease according to Govetto et al. [8] was insufficient for statistical analysis, no group with DRIL was included in the current study (Tables 1 and 2). No difference in cataract severity according to Lens Opacities Classification System III (LOCS III) was found between all groups of ERM OCT morphotypes (Table 1) [16].

**TABLE 1:** The functional outcomes and the distribution of cataract severity in groups of patient eyes with different OCT morphotypes of epiretinal membrane (ERM), with the corresponding significance level (*p*‐value, each ^∗∗^—*p* < 0.01) [16].

OCT morphotypes of ERM		*n*	Age (years)	FECS (%)	BCVA, decimal			LOCS III, units, mean value with standard deviation		
					Preoperative	Postoperative	Difference	Nuclear (*N*)	Cortical (*C*)	Posterior subcapsular (*P*)
Groups	Properties									
Group 1	Preserved foveolar depression	36	76.2 ± 6.5	69	0.43 ± 0.12	0.73 ± 0.27	0.30 ± 0.24	2.7 ± 0.4	1.5 ± 0.5	0.03 ± 0.17
Group 2	Isolated elevation of the foveola	38	75.0 ± 6.1	61	0.38 ± 0.19	0.60 ± 0.23	0.22 ± 0.22	2.7 ± 0.4	1.6 ± 0.5	0.03 ± 0.16
Group 3	EIFL	81	74.7 ± 6.4	70	0.38 ± 0.15	0.69 ± 0.26	0.30 ± 0.27	2.6 ± 0.5	1.5 ± 0.5	0.01 ± 0.14
Group 4	MLH of tractive genesis	31	75.2 ± 6.8	74	0.52 ± 0.15	0.72 ± 0.22	0.20 ± 0.19	2.6 ± 0.5	1.4 ± 0.5	0.00 ± 0.00
Group 5	VMT	15	73.2 ± 7.3	80	0.45 ± 0.17	0.74 ± 0.25	0.29 ± 0.18	2.7 ± 0.5	1.5 ± 0.5	0.07 ± 0.26
Groups 1–3	Without tractional component	155	75.1 ± 6.3	68	0.39 ± 0.16	0.68 ± 0.26	0.28 ± 0.25	2.7 ± 0.5	1.5 ± 0.5	0.02 ± 0.14
Groups 4‐5	With tractional component	46	74.6 ± 6.8	76	0.50 ± 0.16	0.73 ± 0.23	0.23 ± 0.19	2.6 ± 0.5	1.4 ± 0.5	0.02 ± 0.15
Groups 1–5		201	74.9 ± 6.4	70	0.42 ± 0.16	0.69 ± 0.25	0.27 ± 0.24	2.7 ± 0.5, *N* ≤ 3	1.5 ± 0.5, *C* ≤ 2	0.02 ± 0.14, *P* ≤ 1
*p*‐value					*p* < 0.01^∗∗^	*p* > 0.05	*p* > 0.05	*p* > 0.05	*p* > 0.05	*p* > 0.05

*Note:* Percentage of patient eyes with postoperative decimal BCVA ≥ 0.63; MHL, macular hole; VMT, vitreomacular traction; *n*, number of patient eyes.

Abbreviations: BCVA, best corrected visual acuity; EIFL, ectopic inner foveal layers; FECS, functional effectiveness criterion of the surgeries; LOCS III, Lens Opacities Classification System III; OCT, optical coherence tomography.

**TABLE 2:** Correlations between qualitative OCT morphological parameters of the inner foveal layers and visual outcomes in epiretinal membrane (ERM) without and with tractional component, each with the corresponding significance level (*p*‐value, each ^∗∗^—*p* < 0.01).

OCT parameters of the inner retinal layers		Age (years)	OCT morphotypes of ERM without tractional component					OCT morphotypes of ERM with tractional component					All OCT morphotypes
			*n*	FECS (%)	BCVA, decimal			*n*	FECS (%)	BCVA, decimal			FECS (%)
					Preoperative	Postoperative	Difference			Preoperative	Postoperative	Difference	
ONL elevation	Unavailable	74.3 ± 6.8	39	72	0.42 ± 0.11	0.73 ± 0.26	0.30 ± 0.25	15	87	0.54 ± 0.11	0.73 ± 0.22	0.20 ± 0.21	76
	Available	74.7 ± 6.5	116	66	0.38 ± 0.17	0.66 ± 0.26	0.27 ± 0.25	31	71	0.48 ± 0.17	0.72 ± 0.24	0.24 ± 0.18	67
	*p*‐value				0.143	0.125	0.375			0.256	0.877	0.76	

Distortion’s depth of the retinal layers	Unavailable	75.4 ± 6.6	7	43^1^	0.33 ± 0.14	0.62 ± 0.39	0.29 ± 0.32	11	73	0.51 ± 0.13	0.75 ± 0.24	0.24 ± 0.20	61
	RNFL	78.1 ± 2.5	1	100^1^	0.50 ± 0.00	1.0 ± 0.00	0.50 ± 0.00	1	100^1^	0.63 ± 0.00	0.80 ± 0.00	0.17 ± 0.00	100^1^
	GCL	73.6 ± 6.4	25	76	0.43 ± 0.18	0.68 ± 0.27	0.24 ± 0.23	8	100^1^	0.55 ± 0.15	0.80 ± 0.14	0.26 ± 0.16	82
	IPL	75.7 ± 6.2	46	70	0.44 ± 0.15	0.68 ± 0.22	0.24 ± 0.26	9	67^1^	0.51 ± 0.14	0.65 ± 0.28	0.14 ± 0.26	69
	OPL	74.9 ± 6.5	76	64	0.36 ± 0.14	0.67 ± 0.27	0.31 ± 0.25	17	71	0.46 ± 0.18	0.71 ± 0.25	0.26 ± 0.16	66
	*p*‐value				0.008^∗^	0.745	0.556			0.581	0.841	0.632	

RNFL distortion	Unavailable	74.4 ± 6.9	13	62	0.38 ± 0.16	0.63 ± 0.35	0.25 ± 0.30	13	85	0.52 ± 0.12	0.80 ± 0.21	0.29 ± 0.18	73
	Available	75.1 ± 6.5	142	68	0.39 ± 0.16	0.68 ± 0.25	0.28 ± 0.25	33	73	0.49 ± 0.17	0.70 ± 0.24	0.20 ± 0.19	69
	*p*‐value				0.788	0.922	0.791			0.734	0.16	0.194	

RNFL break	Unavailable	73.8 ± 6.2	31	65	0.38 ± 0.15	0.66 ± 0.30	0.28 ± 0.27	21	86	0.50 ± 0.12	0.79 ± 0.19	0.29 ± 0.17	73
	Available	75.4 ± 6.9	124	68	0.40 ± 0.16	0.68 ± 0.25	0.28 ± 0.25	25	68	0.50 ± 0.18	0.67 ± 0.25	0.18 ± 0.19	68
	*p*‐value				0.501	0.982	0.79			0.891	0.103	0.071	

Localization of MFT	temporal	74.8 ± 6.4	33	70	0.38 ± 0.15	0.67 ± 0.27	0.29 ± 0.24	12	75	0.49 ± 0.14	0.74 ± 0.28	0.26 ± 0.25	71
	Central	74.4 ± 6.6	88	65	0.39 ± 0.16	0.66 ± 0.27	0.27 ± 0.27	19	68	0.45 ± 0.18	0.68 ± 0.24	0.23 ± 0.16	65
	Nasal	76.6 ± 6.7	34	73	0.42 ± 0.15	0.71 ± 0.23	0.29 ± 0.22	15	87	0.57 ± 0.10	0.77 ± 0.19	0.19 ± 0.18	77
	*p*‐value				0.416	0.737	0.826			0.055	0.607	0.44	

ERM separation	Unavailable	74.3 ± 6.6	76	68	0.40 ± 0.15	0.69 ± 0.25	0.29 ± 0.25	29	79	0.52 ± 0.13	0.75 ± 0.21	0.23 ± 0.19	70
	Available	75.0 ± 6.5	79	67	0.39 ± 0.16	0.66 ± 0.27	0.26 ± 0.25	17	71	0.46 ± 0.19	0.68 ± 0.27	0.23 ± 0.19	68
	*p*‐value				0.916	0.703	0.685			0.25	0.458	0.631	

*Note:* Percentage of patient eyes with postoperative decimal BCVA ≥ 0.63, IPL/OPL, inner/outer plexiform layer, *n,* number of patient eyes.

Abbreviations: BCVA, best corrected visual acuity, FECS, functional effectiveness criterion of surgeries; GCL, ganglion cell layer; MFT, maximum foveal thickness; OCT, optical coherence tomography; ONL, outer nuclear layer; RNFL, retinal nerve fiber layer.

^1^Percentage result when the number of patient eyes is less than 10.

^∗^ *p* < 0.05.

Qualitative OCT morphological parameters included the following:

1. The RPE is a functional light filter, metabolic supporter of the outer photoreceptor layer, and pump barrier between the choroidal capillary chain and the retina. The changes in the RPE could be divided into three variations: intact (Figure 1), wavy (Figure 4) in drusen, and incipient atrophy in dry age‐related macular degeneration.
2. The ellipsoid zone appears visually in OCT morphology as the second hyperreflective band [17]. Histologically, it appears as the inner segments of the PR, predominantly the cones. The integrity of the ellipsoid zone was divided into intact (Figure 2) and interrupted (Figure 1).
3. The outer limiting membrane (ELM) is, in OCT morphology, the boundary between the inner and outer retinal layers, and histologically, the connection between Müller cell processes and PR. The integrity of the ELM was found in three variations: intact (Figure 1), wavy (Figure 4), and interrupted (Figure 5).
4. Presence of a cotton ball sign (Figure 5), which was described as a hyperreflective, vitelliform, blurred phenomenon between the RPE and the ellipsoid zone (Table 3).
5. Presence of detachment of the outer nuclear layer (ONL) (Figure 2) (nuclei of cones and rods).
6. Depth of distortion of inner foveal layers (Figure 3) due to tangential traction forces, from the retinal nerve fiber layer (RNFL) to the outer plexiform layer (OPL).
7. Presence of RNFL distortion (Figure 1), which plays a crucial role as a collection of third‐order neuron axons, which can be injured during ILM peeling and subsequently thinned [18].
8. Presence of RNFL disruption (Figure 5), which appears as an optical phenomenon in the mid‐OCT section.
9. Location of maximum foveal thickness, temporal, central, and nasal, respectively (Figure 3).
10. The separation of the ERM (adherent (Figure 2) and separated (Figure 4), which could also potentially influence a successful membranectomy (Table 2).
11. The edematous changes of the inner foveal layers could be divided into 3 categories:
  a. Presence of ONL/OPL pseudomicrocysts: microcystic ONL lesions up to 50 μm in size (MME) without the leakage activity described in the literature [19] during fluorescein angiography, as well as cystoid macular edema (CME), each with leakage activity, in cases of foveal distortion‐related Müller cell dysfunction.
  b. Presence of INL pseudomicrocysts (Figure 4).
  c. Location of the pseudomicrocystic foveal lesions: temporal, central, and nasal (Table 4).

Some qualitative foveal parameters are indicated by arrows in Figures 1, 2, 3, 4, 5 with corresponding descriptions.

**TABLE 3:** Correlations between qualitative OCT morphological parameters of the outer foveal layers and visual outcomes in epiretinal membrane (ERM) without and with tractional component, with the corresponding significance level indicated (*p*‐value, ^∗^—*p* < 0.05, ^∗∗^—*p* < 0.01).

OCT parameters of the outer retinal layers		Age (years)	OCT morphotypes of ERM without tractional component					OCT morphotypes of ERM with tractional component					All OCT morphotypes
			*n*	FECS (%)	BCVA, decimal			*n*	FECS (%)	BCVA, decimal			
					Preoperative	Postoperative	Difference			Preoperative	Postoperative	Difference	FECS (%)
RPE lesions	Intact	73.4 ± 6.1	121	76	0.40 ± 0.16	0.71 ± 0.23	0.31 ± 0.23	39	79	0.50 ± 0.16	0.74 ± 0.23	0.24 ± 0.20	77
	Wavy	77.4 ± 4.6	27	44	0.39 ± 0.15	0.59 ± 0.31	0.20 ± 0.30	7	57^1^	0.47 ± 0.17	0.63 ± 0.22	0.16 ± 0.12	47
	Atrophic	78.3 ± 3.8	7	0^1^	0.35 ± 0.19	0.40 ± 0.30	0.05 ± 0.27	0	—	—	—	—	0^1^
	*p*‐value				0.669	0.006^∗∗^	0.011^∗^			0.636	0.218	0.263	

Integrity of ellipsoid zone	Intact	73.5 ± 6.2	86	76	0.38 ± 0.16	0.71 ± 0.25	0.33 ± 0.24	31	87	0.54 ± 0.13	0.76 ± 0.19	0.23 ± 0.17	77
	Interrupted	76.3 ± 5.9	69	58	0.41 ± 0.16	0.63 ± 0.26	0.22 ± 0.26	15	53	0.42 ± 0.18	0.65 ± 0.30	0.23 ± 0.23	57
	*p*‐value				0.318	0.048^∗^	0.034^∗^			0.019^∗^	0.232	0.751	

ELM integrity	Intact	74.3 ± 6.2	110	75	0.40 ± 0.16	0.70 ± 0.25	0.30 ± 0.25	33	82	0.53 ± 0.14	0.75 ± 0.22	0.23 ± 0.20	76
	Wavy	76.8 ± 6.6	38	55	0.39 ± 0.15	0.64 ± 0.29	0.24 ± 0.26	8	75^1^	0.53 ± 0.11	0.73 ± 0.22	0.20 ± 0.16	59
	Interrupted	70.6 ± 8.1	7	29^1^	0.30 ± 0.14	0.46 ± 0.19	0.16 ± 0.15	5	40^1^	0.28 ± 0.13	0.56 ± 0.30	0.28 ± 0.19	33
	*p*‐value				0.296	0.029^∗^	0.149			0.013^∗^	0.354	0.696	

Cotton ball sign	Unavailable	74.2 ± 6.5	120	63	0.37 ± 0.16	0.65 ± 0.27	0.28 ± 0.26	42	74	0.49 ± 0.15	0.71 ± 0.23	0.21 ± 0.19	66
	Available	76.5 ± 6.1	35	83	0.46 ± 0.13	0.75 ± 0.19	0.29 ± 0.24	4	100^1^	0.56 ± 0.19	0.93 ± 0.15	0.37 ± 0.17	85
	*p*‐value				0.002^∗∗^	0.028^∗^	0.684			0.573	0.047^∗^	0.092	

*Note:* Percentage of patient eyes with postoperative decimal BCVA ≥ 0.63, number of patient eyes.

Abbreviations: BCVA, best corrected visual acuity; ELM, external limiting membrane; FECS, functional effectiveness criterion of the surgeries; OCT, optical coherence tomography; RPE, retinal pigment epithelium.

^1^Percentage result when the number of patient eyes is less than 10.

**TABLE 4:** Correlations between qualitative OCT morphological parameters in concomitant macular edema and visual outcomes in epiretinal membrane (ERM) without and with tractional component, each with the corresponding significance level (*p*‐value).

OCT parameters of macular edema		Age (years)	OCT morphotypes of ERM without tractional component					OCT morphotypes of ERM with tractional component					All OCT morphotypes
			*n*	FECS (%)	BCVA, decimal			*n*	FECS (%)	BCVA, decimal			
					Preoperative	Postoperative	Difference			Preoperative	Postoperative	Difference	FECS (%)
ONL/OPL pseudomicrocysts	Unavailable	75.1 ± 6.8	125	70	0.40 ± 0.15	0.68 ± 0.26	0.28 ± 0.26	13	85	0.51 ± 0.13	0.72 ± 0.24	0.22 ± 0.22	71
	Isolated ONL microcysts	77.3 ± 7.2	21	67	0.37 ± 0.17	0.71 ± 0.26	0.34 ± 0.19	9	78^1^	0.47 ± 0.13	0.72 ± 0.21	0.25 ± 0.19	70
	Cystoid macular edema	72.5 ± 7.3	9	44^1^	0.41 ± 0.18	0.54 ± 0.16	0.13 ± 0.23	24	71	0.50 ± 0.18	0.73 ± 0.25	0.23 ± 0.18	64
	*p*‐value				0.849	0.124	0.155			0.692	0.973	0.985	

INL pseudomicrocysts	Unavailable	75.8 ± 6.8	112	71	0.40 ± 0.16	0.69 ± 0.27	0.29 ± 0.25	21	86	0.51 ± 0.16	0.71 ± 0.21	0.20 ± 0.16	74
	Available	72.3 ± 6.5	43	58	0.38 ± 0.16	0.64 ± 0.23	0.26 ± 0.27	25	68	0.49 ± 0.15	0.74 ± 0.26	0.25 ± 0.21	62
	*p*‐value				0.307	0.126	0.684			0.699	0.493	0.4	

Localization of macular edema	Unavailable	75.3 ± 6.9	101	71	0.40 ± 0.15	0.68 ± 0.27	0.27 ± 0.25	7	100^1^	0.53 ± 0.10	0.74 ± 0.14	0.21 ± 0.14	73
	Eccentric	72.3 ± 5.8	18	67	0.33 ± 0.13	0.71 ± 0.23	0.39 ± 0.22	2	50^1^	0.57 ± 0.09	0.60 ± 0.56	0.03 ± 0.47	65
	Central	74.2 ± 6.3	36	58	0.40 ± 0.18	0.64 ± 0.25	0.24 ± 0.26	37	73	0.49 ± 0.17	0.73 ± 0.23	0.24 ± 0.18	66
	*p*‐value				0.107	0.389	0.134			0.707	0.955	0.786	

*Note:* Percentage of patient eyes with postoperative decimal BCVA ≥ 0.63, ONL/INL, outer/inner nuclear layer, *n,* number of patient eyes.

Abbreviations: BCVA, best corrected visual acuity; FECS, functional effectiveness criterion of the surgeries; OCT, optical coherence tomography.

^1^Percentage result when the number of patient eyes is less than 10.

Statistical processing for each group of variations examined was performed using descriptive statistics in IBM SPSS Statistics Version 26. A mean with standard deviation was calculated for the OCT morphological parameters. Statistical significance in the case of the null hypothesis was determined using the *p*‐value, using the Mann–Wittney U and Kruskal–Wallis tests, depending on the number of groups. For this number of patient eyes, a maximum of 15 OCT morphological parameters could be statistically evaluated. Nevertheless, the significance level for the null hypothesis was set at *p* < 0.005 after the Bonferroni correction to avoid type 1 statistical errors. In addition, a functional effectiveness criterion (FECS) for the combined procedure was introduced. This is the percentage of patient eyes achieving a postoperative decimal BCVA ≥ 0.63, which corresponds to the requirements of the German ophthalmological road traffic assessment for Class B in functional monocular vision. The FECS cumulatively represents the possibility for patients to maintain a relatively unrestricted professional and private quality of life after surgery. For each qualitative OCT morphological parameter examined, the mean patient age with standard deviation was also determined.

## 3. Results

As shown in Table 1, a significant difference in preoperative BCVA was found between all OCT morphotypes of ERM. This difference was even more significant with respect to the presence of the tractional component (Table 1). It is noteworthy that preoperative BCVA was significantly higher in the ERM groups with a tractional component. At the same time, no difference in postoperative BCVA was observed across all OCT morphotypes of ERM, including those with the tractional component. Approximately two‐thirds of patient eyes benefit from combined surgery in terms of postoperative visual outcomes. Our data are consistent with the data of other authors [20]. In the case of VMT, this ratio even increased to four‐fifths of successfully operated patient eyes.

Table 3 shows the correlations between qualitative OCT morphological changes in the outer foveal layers and visual acuity results. In the OCT morphotypes of ERM without a tractional component, no correlations were found between preoperative visual acuity results and RPE lesions or the integrity of the ellipsoid zone and ELM. In contrast, postoperative BCVA and the difference in visual acuity correlated negatively with the above‐mentioned preoperative OCT parameters. Surprisingly, the presence of the cotton ball sign correlated positively with both preoperative and postoperative BCVA. This phenomenon may be explained by the earlier onset of functionally reversible lesions compared to irreversible morphological damage. A different picture was observed in the presence of a tractional component. Only the integrity of the ellipsoid zone and the ELM positively influenced the preoperative visual acuity results and played no role in postoperative visual acuity prognosis. Only the better postoperative BCVA was shown in cases of OCT morphologically evident cotton ball sign, as well as in cases of absence of traction component.

However, a significant difference in FECS of over 20% was found for all lesions of the outer foveal layers, suggesting that these could serve as an indirect marker of poor postoperative visual prognosis in all OCT morphotypes of ERM.

As shown in Table 2, almost all changes in the inner foveal layers in all OCT morphotypes of ERM correlated neither with preoperative nor with postoperative BCVA. This hypothesis was also confirmed using FECS, where no significant difference was found for the inner foveal layer lesions, excluding macular edema.

Exceptions to this were the negative correlations between preoperative BCVA and the depth of retinal layer distortion in ERM without a tractional component.

Regarding the OCT morphological signs of macular edema (Table 4), no significant correlation was found between the individual characteristics of microcyst location and size and the visual outcomes. It is noteworthy that the FECS in MME in the ONL is significantly higher in OCT morphotypes of ERM without a tractional component compared to those with CME. The location of macular edema also played no role in the postoperative visual acuity prognosis. In view of the patient age in relation to the qualitative OCT morphological parameters, no trends or significant correlations were found except for age‐related RPE changes in ERM.

## 4. Discussion

This study demonstrated several positive correlations between qualitative changes in the individual foveal layers and visual acuity outcomes, which was also demonstrated for certain markers of postoperative visual acuity prognosis. Some of the latter have also been investigated by other authors, with their respective significance determined. In the review by Fung et al. [5], a good visual acuity prognosis was associated with the absence of EIFL, CME, cotton ball sign, and ellipsoid zone defects. These findings were partially consistent with our results regarding ellipsoid zone and CME lesions in ERM without a traction component, but they are controversial regarding changes in the inner foveal layers (EIFL) and the cotton ball sign. As described, no significant difference in postoperative BCVA or significant FECS differences were found between all OCT morphotypes, including those with EIFL.

A significant difference in preoperative BCVA was found between all OCT morphotypes, including those with EIFL, which is consistent with the study by Govetto et al. [8] in 194 patient eyes vitrectomized for ERM with progressively stage‐dependent decreased BCVA (*p* < 0.001). In contrast to other studies [5], in our study, patients benefited in terms of postoperative visual outcome in the presence of the cotton ball sign.

The disintegrated ellipsoid zone played a decisive negative role in the postoperative visual prognosis in OCT morphotypes without a tractional component, which has also been described in other studies [7, 13, 21–23]. This was also the case in the study by Inoue et al. [14], which included a total of 45 patient eyes with idiopathic ERM that underwent vitrectomy. A significantly better postoperative BCVA was shown with an intact IS/OS junction compared to an interrupted IS/OS junction (*p* < 0.001).

Following the analysis of 79 vitrectomy‐treated eyes with symptomatic ERM in the study by Sheales et al. [22], no significant visual prognostic factors were detected in terms of retinal contraction and the presence of intraretinal microcysts. These results are fully consistent with our findings. However, in contrast to this study, we found that ELM integrity in OCT models of ERM without a tractional component significantly correlated with both preoperative and postoperative BCVA.

The study by Kim et al. with 43 pseudophakic patient eyes aged 64.88 ± 10.46 years who underwent vitrectomy for idiopathic ERM showed that changes in the inner retinal layers were associated with a better visual prognosis (*p* < 0.001) than lesions in the outer retinal layers [24]. This hypothesis contradicts the data we collected, which showed that lesions of the inner foveal layers played almost no role in postoperative visual acuity prognosis. This difference could be due to the fact that patients with stage 4 according to Govetto et al. [8] were not included in the current study. Further studies are needed to clarify the discrepancy in our data.

In the study by Guber et al. [25] with 36 vitrectomized patient eyes with LMH, a significant improvement in BCVA (LogMAR) from 0.3 to 0.2 was shown at 3 months postoperatively. These results correlate with our results, which showed a preoperative decimal BCVA of 0.52 ± 0.15 and a postoperative decimal BCVA of 0.72 ± 0.22 (Table 1).

Regarding MME, full agreement was found with the studies by Govetto et al. [19] with 123 patient eyes and Yang et al. [26] and Lee et al. with 100 patient eyes. It was demonstrated that MME could not serve as a prognostic factor in ERM.

The study by Doguizi et al. [27] showed a significant correlation of the following OCT morphological parameters, such as EIFL, MME, cotton ball signs, and the disintegrity of the ellipsoid zone, with preoperative BCVA, which also corresponds to the data from our study excluding MME.

The study by Hsia et al. [28] demonstrated the influence of changes in the inner foveal layers on visual acuity after analyzing 600 OCT findings from the eyes of patients with ERM. In our study, similar results were observed with regard to the correlations between the OCT morphotypes according to Govetto et al. [8] and preoperative BCVA.

## 5. Conclusions

This retrospective study shows that the overall OCT morphology of ERM and the presence of a tractional component significantly influence preoperative visual acuity outcomes. At the same time, these components play no role in postoperative visual acuity assessment. OCT morphological analysis of the individual foveal layers revealed clear markers of poorer postoperative visual prognosis in ERM without a tractional component. In the presence of a tractional component, no reliable OCT morphological factor for visual prognosis was found in either the outer or inner foveal layers, except for the presence of a cotton ball sign, just as in OCT morphotypes of ERM. The changes in the inner foveal layers did not correlate with either preoperative or postoperative BCVA.

The exception to this was the negative correlation between preoperative BCVA and the depth of retinal distortion in the ERM groups without a tractional component. Likewise, no correlation was found between macular edema and visual outcomes, either with or without a tractional component. The exception to this was the presence of CME in OCT morphotypes without a tractional component, which was associated with poor functional postoperative outcomes.

**Nomenclature**
BCVA: Best corrected decimal visual acuity
DRIL: Disorganization of the inner retinal layers
EIFL: Ectopic inner foveal layers
ELM/ILM: Outer/inner retinal limiting membrane
ERM: Epiretinal membrane
FECS: Functional effectiveness criterion of surgery
IOL: Intraocular lens
LMH: Lamellar macular hole
MME/CME: Microcystic/cystoid macular edema
OCT: Optical coherence tomography
OI/OS junction: Junction between the inner and outer segments of the photoreceptors (PRs)
ONL/INL: Outer/inner retinal granular layer
OPL/IPL: Outer/inner retinal plexiform layer
RNFL: Retinal nerve fiber layer
RPE: Retinal pigment epithelium
VMT: Vitreomacular traction

## Funding

The authors received no specific funding for this work. Open access funding enabled and​ organized by Projekt DEAL.

## Disclosure

All authors agree with the publication of this paper.

## Ethics Statement

All the patients signed informed consent preoperatively regarding the processing of their findings, after a detailed explanation. This corresponded to the contemporary requirements of the local Ethics Committee regarding retrospective studies. All the patient‐relevant principles of the Declaration of Helsinki were also strictly adhered to.

## Conflicts of Interest

The authors declare no conflicts of interest.

## Data Availability

The authors are ready to provide anonymized primary data at any time upon request.
